# Supplementary material for: Gut Microbiota Differentially Mediated by Qingmao Tea and Qingzhuan Tea Alleviated High-Fat-Induced Obesity and Associated Metabolic Disorders: The Impact of Microbial Fermentation
Source: Foods. 2022 Oct 14;11(20):3210. doi: 10.3390/foods11203210 (PMC9601715; doi:10.3390/foods11203210)
Supplement: Supplementary file 1 [file foods-11-03210-s001.zip › foods-1928758-supplementary.pdf]

Supplementary Materials

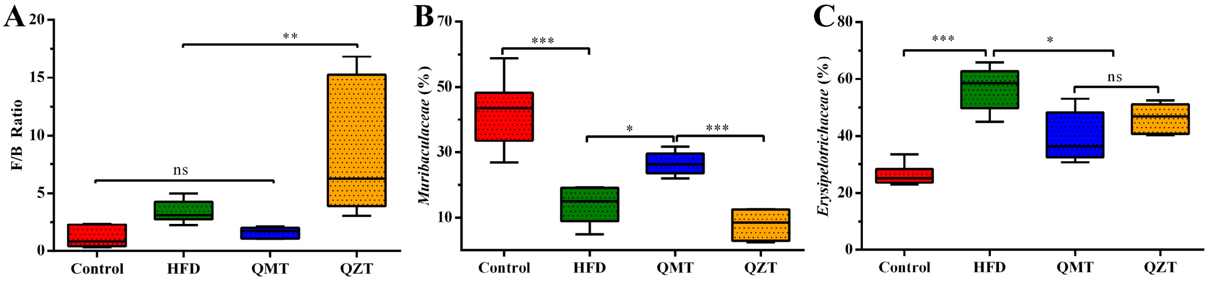

Figure S1. (A) The F/B ratio, and the relative abundance of *Muribaculaceae* (B) and *Eyrsipelotrichaceae* (C) among the control, HFD, QMT, and QZT groups.

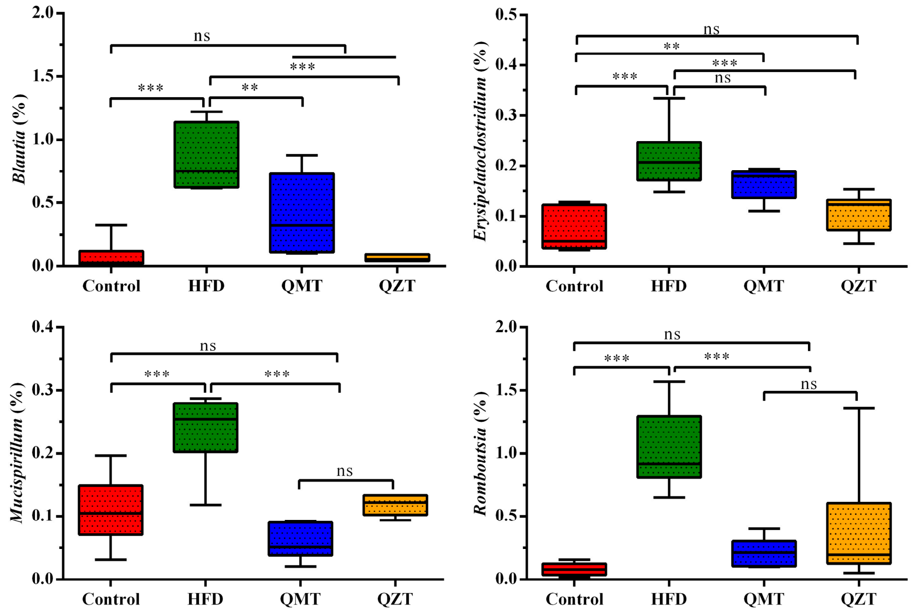

Figure S2. The gut microbiota restored by QMTe and QZTe treatment at the genus level.
